# Bagasse minority pathway expression: Real time study of GH2 β-mannosidases from bacteroidetes

**DOI:** 10.1371/journal.pone.0247822

**Published:** 2021-03-17

**Authors:** Tatiane Fernanda Leonel, Elisângela Soares Gomes Pepe, Tereza Cristina Luque Castellane, Juliana da Silva Vantini, Michelli Inácio Gonçalves Funnicelli, Eliana Gertrudes de Macedo Lemos

**Affiliations:** 1 School of Agricultural and Veterinarian Sciences, São Paulo State University (UNESP), Jaboticabal, SP, Brazil; 2 Department of Technology, Laboratory of Biochemistry and Plant Microorganisms, Jaboticabal, São Paulo, Brazil; 3 Graduate Program in Agricultural and Livestock Microbiology, School of Agricultural and Veterinarian Sciences, São Paulo State University (UNESP), Jaboticabal, SP, Brazil; Kyungpook National University, REPUBLIC OF KOREA

## Abstract

After being isolated from a sugarcane pile, the bacterium *Chitinophaga* sp. CB10 demonstrated to be a rich source of carbohydrases, with 350 predicted CAZyme domains. CB10 was able to grow on carbohydrates of different structural complexities: glucose, carboxymethylcellulose, corn starch, galactomannan, *Aloe vera* gum and sugarcane bagasse. The sugarcane bagasse is a rich source of complex polymers, and the diversity of metabolites released by its enzymatic hydrolysis has an important role for green chemistry, including minority pathways such as the degradation of mannan conjugates. In this sense, CB10 demonstrated considerable levels of gene expression for mannanases, and was stable for a period of 96–144 hours in the presence of sugarcane bagasse as sole carbon source. The bacterium showed respectively 4.8x and 5.6x expression levels for two genes predicted for GH2 β-mannosidase: one located within a gene cluster identified as “polysaccharide utilization loci” (PUL), and another a classic β-mannosidase. These enzymes shared less than 45% of identity with enzymes characterized from the genus *Chitinophaga* belonging to the phylum Bacteroidetes. The degree of novelty—as demonstrated by the low identity with previously characterized enzymes; the remarkable capability to grow in different substrates; mannanase activity, evidenced by the release of residual oligosaccharides in the cultivation with galactomannan (HPLC-RID, 12.3 mMol); associated to the ability of mannanases expression in a low concentration of inductor conditions (sugarcane bagasse, 0.2%) indicate the high potential for the application of CB10 as a source of enzymes in the production of oligosaccharides from biomass. This capacity might prove to be very valuable for the biorefinery process of pre-biotic precursors and other functional oligosaccharides focused on the food and pharmaceutical industries.

## Introduction

The phylum Bacteroidetes is abundant in many habitats, and some species in this group can hydrolyse a wide variety of complex carbohydrates, including the use of non-conventional polysaccharide degradation systems known as polysaccharide utilization loci (PUL). PUL are clusters of genes peculiar to this phylum, specialized in the effective degradation of different carbohydrates [1–8]. A bacterial *Chitinophaga* sp. CB10 from the bacterial consortium of sugarcane bagasse was previously isolated [9] and the subsequent annotation of its partial genome enabled the identification of 350 CAZyme domains involved in biomass degradation: 202 GHs, 77 GTs, 44 CEs, 33 CBM, 16 PL and 7 AA. More of the 30 PULs-pathways were identified for this genome, and 27% of its annotated GHs were found in PUL-like clusters.

A recent proteomic study of a strain of *Chitinophaga pinensis* (UQM 2034T) Sangkhobol and Skerman 1981 [10], that belongs to the order Chitinophagales and the Chitinophagaceae family (NCBI: txid 79329), was conducted in the presence of mannose-conjugated substrates, and several of the predicted enzymes related to the PUL system were identified [8]. The mannans are widely distributed in plants as structural polymers and reserve non-starch carbohydrates. They are found in many organism tissues, such as the hemicellulose from wood cell walls (mainly soft wood); endosperm, seed vacuoles and the mucilage of *Aloe vera* (L.) Burm. F. Bradley 1992 [11], belongs to the order Liliales and family Aloaceae (NCBI: txid 34199), as well as in red and green algae [12, 13]. They are organized in different combinations of mannose and other sugars, creating four types of main structures: pure mannan (which may contain less than 5% of other sugars), glucomannan, galactomannan and galactoglucomannan. Some mannose combinations can also have unusual modifications, such as sulfation, as found in the mucilage of the red alga *Nothogenia fastigiata* (Bory) P.G. Parkinson 1983 [14], which belongs to the order Nemaliales and family Scinaiaceae (NCBI: txid 268581), or acetylations found in *Aloe vera* carrysin [12, 15]. Mannnose may also be present in other polymers as a secondary component, as can be seen in sugarcane bagasse hemicellulose, predominantly constituted by L-arabino-(4-O-methyl-D-glucurono)-D-xylan, but containing small traces of the sugars D-mannose, L-rhamnose and D-galactose associated with the xylan backbone [16].

Due to the complex and heterogeneous nature of mannans and other mannose conjugates, several synergistic acting enzymes are needed for their hydrolysis. The enzymes involved in the hydrolysis of different types of mannan polymers are collectively called mannanases. Among these are the endo-acting 1,4-β-D mannan mannohydrolase (β-mananase, E.C 3.2.1.78); the exo-acting 1,4-β-D-mannopyranoside hydrolase (β-mannosidase, E.C 3.2.1.25) and the β-glucosidase (1,4-β-D-glucoside glucohydrolase, EC 3.2.1.21), which act in the main backbone-chain cleavage; whereas accessory enzymes, such as acetyl mannan esterase (EC 3.1.1.6) and α-galactosidase (1,6-α-D-galactoside galactohydrolase, EC 3.2.1.22) act in the side-chain breakdown [12, 15, 17]. Among these enzymes, β-mannosidase has an essential role in oligosaccharides processing by attacking the terminal ends at the non-reducing end, as well as cleaving mannobiose into mannose units [15, 18].

β-mannosidases are produced by several organisms such as plants, animals, fungi and bacteria [15] (NCBI <https://www.ncbi.nlm.nih.gov/>). In plants, they have an essential role in the mechanism of use of endosperm reserves during seed germination, as well as being responsible for the production of oligosaccharides that act in signalling the modulation of plant growth and development [12, 18]. Amongst microbial enzymes, the diversity of β-mannosidases is immense, being grouped in the families of GH1, 2, 5 and 164, based on characteristics in their amino acid sequences, structural similarities and mechanisms of action [19–22]. Considering the great potential for the application of mannanases as β-mannosidases in food, detergents, fuel, feed, and paper and cellulose industries, these enzymes, especially the microbial ones, which hold the greatest diversity, have attracted the attention of researchers in the search for viable and effective alternatives for large-scale use [12, 18]. However, β-mannosidases are still relatively poorly explored when compared to the intense use of other carbohydrases, such as cellulases and xylanases [18]. Nevertheless, some studies have shown that microbial mannanases can act in a wide range of pH (3–7.5) and temperature (45–92°C), which is highly desirable for several industrial applications [12, 15, 18, 23, 24].

Unlike what happened recently with cellulose, the degradation of mannan polymers for the application in the production of biofuel from lignocellulosic biomass has not yet become a sustainable process, even when using raw materials rich in mannose, such as softwoods like conifers. It is therefore rarely used as a substrate for fermentation, although, not long ago, a research focusing on the production of yeasts that were efficient for mannan degradation tried to change this scenario [13]. In spite of this, the amount and diversity of vegetal residues that contain mannose polymers, such as coffee seed residues, palm kernels and copra meals, are the target of a great deal of interest for biorefinery [13, 18]. In this sense, bioconversion technologies using enzymes are rapidly emerging as a solution for the manufacturing of products with high added value [15]. Among these applications, one that has recently attracted significant interest is the use of β-mannosidases in the production of manooligosaccharides with the potential application as prebiotics and dietary fibers [18, 25, 26]. Another example of application is the use of manooligosaccharides obtained from *Aloe vera* as a protective agent against the effects of ultraviolet light (patent WO1998009635). In addition, the linear acetomannan of *Aloe vera* (carrysin) has immuno-pharmacological and therapeutic properties [12], which can be amplified by punctual changes in the mannose structure by mannanases action.

Although abundant in many other plants biomass, the content of mannose is not very significant in sugarcane bagasse hemicellulose (0.2%) when compared to the content of xylan (24.7%) or even arabinan (1.6%) [27]. Thus, the low content of mannan in this substrate can lead to a simplistic view that the pathways involved in the degradation of mannan in sugar cane biomass can be neglected, though oligosaccharides of different complexities can be obtained through biomass biorefinery. Nevertheless, these minority compounds seem to be of importance from a physiological point of view, since microorganisms isolated by our team from communities associated with the degradation of substrates that are poor in mannan often have several mannanases in their genetic arsenal [9, 28, 29].

Thus, we present a comprehensive study of the real-time expression in the sugarcane bagasse of two β-mannosidases belonging to the GH2 family: CB10-153.446 and CB10-00347, both prospected and submitted to functional annotation by homology of the genome of the *Chitinophaga* sp. CB10. This genus is known for its extensive ability for enzymatic degradation of complex polysaccharide materials, acting on carbon cycling in its natural environment. These enzymes can be used in biotechnological processes to deconstruct the plant cell wall, leading to the release of hemicellulose fractions containing mannan groups that have potential applications in the synthesis of oligosaccharides for the production of biofuels, prebiotics, drugs and other biocompounds. Our main objective is to provide theoretical and practical discoveries that contribute to the elucidation of the functioning of the genetic machinery of this bacterium in mannan degradation, reinforcing its degradation capacity and the benefits of applying these enzymes in the deconstruction of plant biomass, albeit in biomass that is very poor in mannan content, such as the sugarcane bagasse.

## Material and methods

This study was carried out with the bacterium *Chitinophaga* sp. CB10 derived from the culture bank at the Laboratory of Biochemistry of Microorganisms and Plants (LBMP) at FCAV/UNESP. The substrates and reagents used were purchased from Sigma Aldrich.

### Prediction Polysaccharide Utilization Loci (PUL)

The GH2 sequences described in this study were identified in previous studies. The annotation of predicted proteins from *Chitinophaga* sp. CB10 was performed with the dbcan2 tool [30] including the dbCAN CAZyme domain (by HMMER search), short conserved motifs (by Hotpep search), and CAZy databases (by DIAMOND search), using default parameters against the CAZY Database (version 2019). The prediction of putative PUL was proceeded by the CGC-Finder [31] carried out with the dbcan tool. The CAZyme gene clusters (CGCs) are annotated based on signature genes related to (i) CAZymes using the CAZy database [32]; (ii) Transporter Classification (TCs) was searched against TCDB [33], and Transcription Factors (TFs) searched against CollectTF [34], DBTBS [35] and RegulonDB [36]. The annotation for CGCs was performed using the dbcan2 tool [30].

### Phenetic analysis

The multiple alignment of the protein sequences in this study with the sequences of the GH2 family featured in the CAZy database [32], and not characterized in the BRENDA database [37], were performed by T-Coffee [38] using the mcoffee mode. Phenetic inference was conducted employing a neighbor-joining method applying the Poisson model and pairwise deletion with 10000 bootstrap replicates, using the MEGAX software [39].

### Cultivation conditions for the bacterial development curve and real-time quantitative PCR analysis (RT-qPCR)

Liquid BHB culture medium (0.2 g L^-1^ MgSO_4_·7H_2_O; 0.02 g L^-1^ CaCl_2_ 2H_2_O; 1.0 g L^-1^ K_2_HPO_4_; 1.0 g L^−1^ KH_2_PO_4_; 1.0 g L^−1^; NH_4_NO_3_; 0.05 g L^−1^; FeCl_3_; 10.0 g L^−1^ Glucose pH 7,0) [40] was used for CB10 cultivation as expression negative control (GLU), while a solid BHB culture medium, replacing the carbon source Glucose per 10.0 g L ^-1^ of washed, dried, crushed and sifted sugarcane bagasse (32 mesh, 0.5mm), and solidified by the addition of agar nutrient (10.0 g L^−1^), was used for the Bagasse treatment (BAG). For the growth curve, the cultures containing the bacteria were incubated in an orbital shaker (30°C/150 rpm) for a period of 0, 2, 4, 6, 24, 48, 72, 96, 120 and 144 hours. The number of viable cells was evaluated in a solid medium after preliminary cultivation in a liquid medium containing bagasse, by colony forming units (CFU), and in a liquid medium by optical density OD_600_ for the control treatment (glucose). For real-time PCR analysis (RT-qPCR), the bacterium was grown as described above, in a liquid culture media. Aliquots were taken for RNA extraction at specific times: 24, 96 and 144 hours, to cover all stages of bacterial development.

### Cultivation conditions for growth analysis of CB10 bacteria on different substrates

To measure sugars released by the DNS and HPLC techniques, the CB10 bacterium was grown in a BHB liquid medium, as described in the previous item, replacing Glucose (10.0 g L^−1^) with different carbon sources (10.0 g L^−1^): Sugarcane bagasse (BAG); Galactomannan (Gum Locust bean) (GAL); and *Aloe vera* (ALV) for 96 hours (30°C/150 rpm).

The capacity of radial development of the bacterial colony in the solid extract was evaluated in a similar way, using the solid culture medium BHB for the same substrates evaluated in the liquid medium, plus the treatments Carboxymethylcellulose (CMC) and Starch (STA). After inoculation, the plates were incubated for 96 hours in B.O.D. at 30°C. Degradation and assimilation capacities were observed through the formation of the colony. The hydrolysis halos were revealed by the application of Congo Red (1%) for 15 minutes, followed by washing the plates with NaCl (1M) for 10 minutes [41].

### High Performance Liquid Chromatography (HPLC)

The analysis of the chromatographic profile of residual sugars from the bacterial metabolic activity on different sources of carbon in the culture medium was performed using the high-performance liquid chromatography (HPLC) technique. After the cultivation of the CB10 bacterium, as described in the previous item, 1 mL aliquots were subjected to centrifugation (Sorvall centrifuge at 16,266 xg for 30 minutes at 4°C), after they were filtered (Millipore filter, 0.22 μm membrane). Then the samples were injected into the HPLC system equipped with a RID detector (Shimadzu, model RID-10A). Aliquots of 10 μl were injected and eluted in a mobile phase of acetonitrile, i.e., water (75:25, vol: vol) under the following chromatographic conditions: injection temperature of 35°C, flow of 1.0 ml/minute. As a negative control, the culture medium was used at zero time for each condition of carbon source. For quantification, standard solutions of 100 μM of each standard sugar (mannose, glucose) were prepared.

### Analysis of reducing sugar by 3,5-dinitrosalicylic acid (DNS)

The analysis of residual reducing sugars from the bacterial metabolic activity on different carbon sources was carried out through the analysis of reducing sugars by 3,5-dinitro-salicylic acid (DNS) as described by Miller [42]. After cultivation described in the item Cultivation conditions for growth analysis of CB10 bacteria on different substrates, the cultures were centrifuged (Sorvall centrifuge at 16,266 xg for 30 minutes at 4°C) and a 100 μL aliquot of the supernatant, plus the same proportion of the DNS reagent, was incubated in the temperature cycling apparatus PCR type Thermo scientific Bio-Rad PTC-100® termal cycler (95°C, 5 min; 4°C, 1 min.; and 20°C, 10 min.). After the reaction, the absorbance was read at 540 nm in a Thermo Scientific™ Multiskan™ GO Microplate Spectrophotometer. The measured absorbance was compared to the values of a standard mannose curve (180.2 mw) at 0 to 20 mM to detect the presence of reducing sugars released in each culture.

### Real-time PCR experiment (RT-qPCR) to evaluate real-time gene expression of mannosidase genes

In order to carry out the evaluation of gene expression in real time (RT-qPCR), it was necessary to design the ORF primer candidates for β-mannosidases, from the sequences (S1 File) obtained from the sequenced partial genome of the CB10 bacterium deposited in the NCBI database accession number: MLAV00000000 [9]. The sequences were confirmed in databases (NCBI: http://www.ncbi.nml.nih.gov/) and aligned with the Bioedit program [43] using the ClustalX 1.5b tool [44]. The primer pairs were designed with the Primer3Express program (PrimerExpress software, Applied Biosystems, version 7500) following the parameters described by Schmittgen [45]. For endogenous "maintenance" control between candidate genes, Sigma A rRNA (primary sigma factor of RNA polymerase) was selected and identified as the most stable and suitable of all software. After designing the primer pairs, the RNA extraction process was carried out with the RNA isolation kit (Mini RNA spin, Synapse biotechnology) following the manufacturer’s instructions. The bacteria were cultured as described in the previous item, and 2 ml aliquots of the cultures were centrifuged (10,000 × g, at 22° C for 10 minutes) to obtain the bacterial pellet. This was resuspended in 100 μL of lysis solution (0.2 mg mL− 1 lysozyme in 1 mL TE) and preheated to 37° C for 10 minutes in Eppendorf ThermoMixer® F1.S. RNA integrity was assessed using the Agilent 2100 Bioanalyzer device and the concentration was assessed using the Qubit® fluorometer kit (Thermo Fisher Scientific) according to the manufacturer’s instructions. To quantify the expression of the RT-qPCR gene in real time, RNA synthesis in cDNA was performed, following the manufacturer’s instructions for the SuperMix SuperScript ™ III First-Strand Synthesis kit (Invitrogen). The concentration of 500 ng of total RNA, 50 ng/μL of random hexamer primers (Applied Biosystems, France) and 200 U of Superscript III reverse transcriptase (Invitrogen, France) in a final volume of 20μL [45] were used. Before performing the RT-qPCR reaction in real time, standardizing the primer pairs was required, as described in Table 1. For each gene, primer concentration standardization tests (100/100nM; 300/300nM and 600/600nM) and the standard cDNA the curve was performed at the following concentrations: 200 ng; 100ng; 50ng; 25ng; 12.5 ng; 6.25 ng and 3.12 ng; totalling seven points. Quantitative PCR was performed using Applied Biosystems 7500 Real-Time version 1.3 (Copyright 2001-2004-Applied Biosystems). The amplification reactions of β-mannosidases were performed with cDNA at a concentration of 130 ng; 6.5 μL of SYBR Green (Thermo Fisher Scientific) 1.25 μL of each primer (F and R) in the following concentrations: 100/100nM for the CB10-153.446 gene, 300/300nM for CB10-00347 and 100/100nM for Sigma One gene, in a final reaction volume of 12.5 μL (ultrapure water). The PCR amplification program consisted of an initial denaturation (94°C/5 min); followed by 35 amplification cycles (94°C/40 sec; 56°C/1 min; 72°C/45 sec) and a final extension (72° C/7 min).

**Table 1:** Characteristics of the primers used in the analysis of RT-qPCR.

Gene name	Name (ORF)	Primer Sequence (5’– 3’)	Lenght (bp)	Tm (°C)	Primer efficiency (%)	R^2^	Slope
β-mannosidase	CB10 00347	**F:**CCGGTGAATAAAATCGTCA	127pb	57.15	106.8	0.99	-3.168
		**R:**TTGCCTCCACGAATAATTG					
β-mannosidase	CB10 153.4461	**F:**TTCCACTCTTCTCCCATCA	102pb	57.15	92.3	0.994	-3.520
		**R:**CGCTTCAAACCATTCCATA					
Sigma A rRNA	CB10 04158	**F:**CGCCAACTCAAAATCACTAA	83pb	58.06	90.7	0.994	-3.567
		**R:**TCCACTTTCCCAATCTCCT					

**Notes: F, forward primer; R, reverse primer, Tm, melting temperature, R**^**2**^ **coefficient of determination, bp, base pairs.**

### Data analysis and relative quantification study

For the analysis of the data generated by the relative quantification of the levels of gene expression, the quantification method (threshold cycle) based on Algorithm 2^−ΔΔCt^ was used, which generates the expression value of the normalized target gene for the endogenous calibrator [46].

All other determinations were carried out with the biological triplicate and the results presented as mean values plus the respective standard deviations.

## Results

### CB10 β-mannosidases annotation: conventional versus Pul-like associated GH2 CAZymes

**Fig 1:**
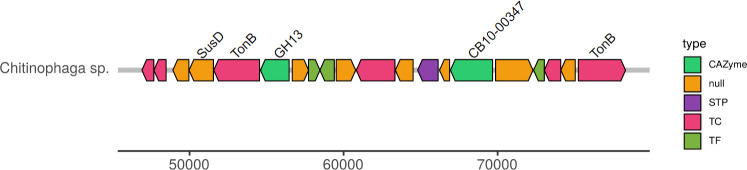
Annotation of genes clustered in “PULs-like” associated with CAZyme GH2 β-mannosidase CB10-00347, obtained from *Chitinophaga sp*. CB10. The annotation was performed using the CGC-Finder program. The GH2 sequences identified in previous studies (unpublished data) were annotated using the dbcan2 tool [30]. The prediction of putative PUL was proceeded by the CGC-Finder [31]. The CAZyme gene clusters (CGCs) are annotated based on related signature genes (i) CAZymes using CAZy database [32]; (ii) Transporter Classification (TCs) were searched against TCDB [33], and Transcription Factors (TFs) searched against CollectTF [34], DBTBS [35] and RegulonDB [36]. The annotation for CGCs was performed using the dbcan2 tool [30]. Legend: CAZyme, “Carbohydrate-Active Enzymes”; null, non-annotated; STP, signal transduction proteins; TC, Transporter classification; TF, Transcription Factor.

Two predicted GH2 β-mannosidases were annotated functionally by homology and prospection for conserved domains from the sequenced genome of *Chitinophaga* sp. CB10, a gram-negative bacterium belonging to the Bacteroidetes phylum, isolated from a lignocellulosic biomass degrading consortium [9]. The annotated β-mannosidases (CB10-153.446 and CB10-00347) shared less than 27% of identity with each other (query coverage of 58%, Blastp, https://blast.ncbi.nlm.nih.gov/Blast.cgi). CB10-00347 was annotated within a PUL gene complex (Fig 1, S3 File), while CB10-153.446 did not show any association with specific enzymatic clusters.

### CB10 β-mannosidase: GH2 CAZymes closest to Bacteroides spp.

With the purpose of targeting a better characterization, CB10 β-mannosidases were compared to two data sets: that of “Cazy β-mannosidases”, corresponding to functionally characterized enzymes identified according to the GH2 group, obtained from the Cazy Database (http://www.cazy.org/); and the “Brenda β-mannosidases”, corresponding to functional predicted sequences from the Brenda Database (https://www.brenda-enzymes.org/). As a result, the CB10 β-mannosidase showed a high indication of novelty: CB10-153.446 shared 43.59% (99% query coverage) with a β-mannosidase from *Bacteroides thetaiotaomicron*, while CB10-00347 shared only 27.93% (91% query coverage) with a β-mannosidase from *B*. *xylanisolvens*.

**Fig 2:**
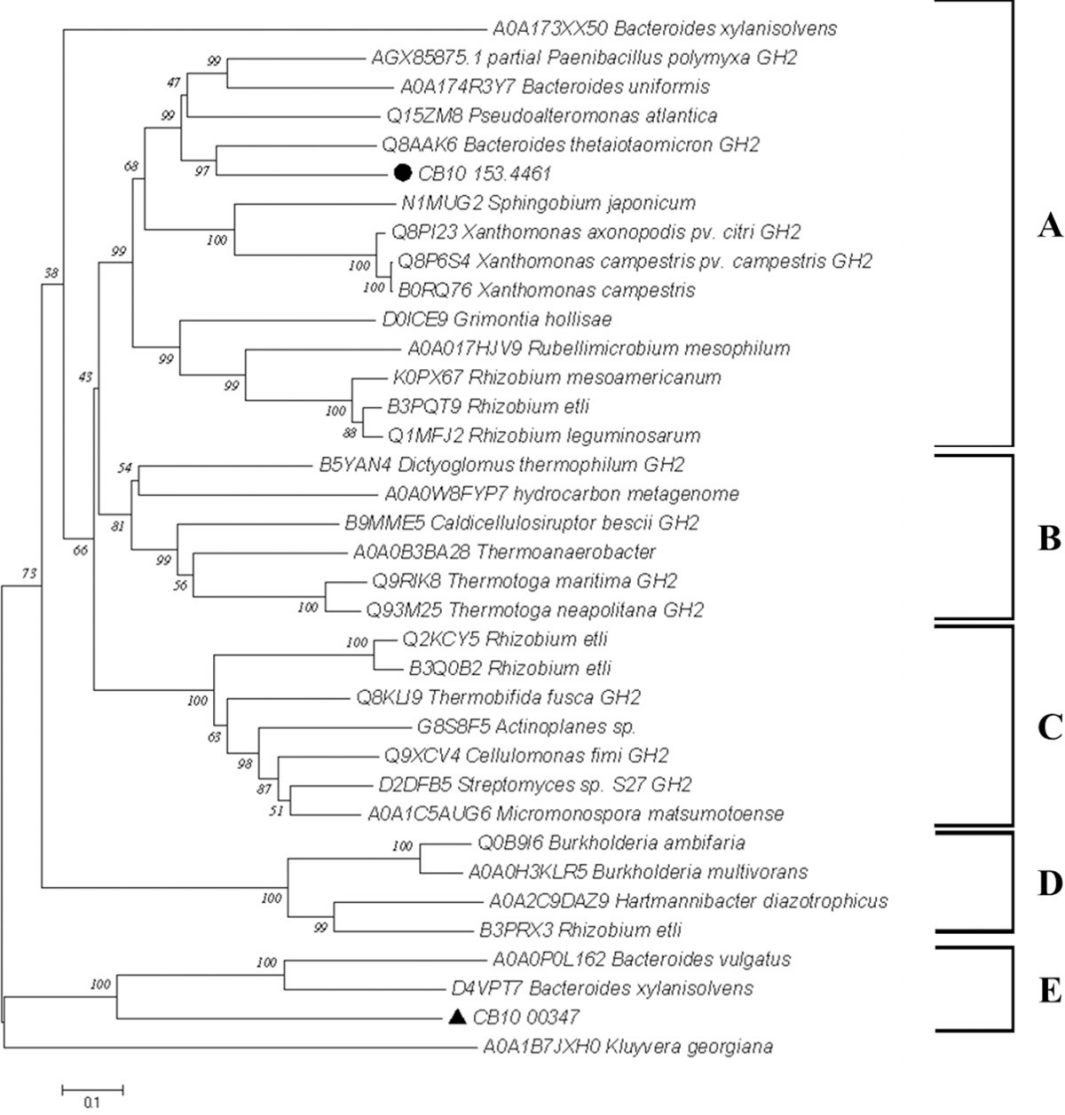
Phenetic dendrogram generated by the MEGA 5 program for β-mannosidases. The analysis was carried out based on the distance matrix generated from an alignment made by the T-coffee, using the Neighbor Joining clustering optimization method [47]. The percentage of replicate trees in which the associated taxa clustered together in the bootstrap test (10,000 replicates) is shown next to the branches [48]. The tree is drawn to scale, with branch lengths in the same units, based on similarity; distances were computed using the Poisson correction method [49] and are in the units of the number of amino acid substitutions per site. The analysis involved 36 amino acid sequences. All positions containing gaps and missing data were eliminated. There was a total of 330 positions in the final dataset. The tree was conducted in MEGA7 [39]. Circle and Triangle: respectively, β-mannosidases CB10-153.446 and CB10-00347.

With the aim of visualizing the distribution by groups of greatest similarity for the sequences, these were submitted to a phenetic analysis, and the experimentally characterized Cazy sequences were used as standards. The multiple alignment with some high conserved blocks was revealed in (S3 File). The result of the phenetic tree is shown in Fig 2, where it is possible to see that most of the sequences were grouped into five clades. Although they were grouped in different clades (Clades **A** and **E**), the two putative β-mannosidases sequences of CB10 were grouped with the sequences that had already returned showing greater similarity with the Blastp analysis, corresponding to the β-mannosidases of bacteria of the genus *Bacteroides* (phylum Bacteroidetes). The sequence of CB10-153.446 grouped together with the largest clade (Clade **A**), next to the most similar sequence of β-mannosidase BtMan2A from *Bacteroides thetaiotaomicron* (Q8AAK6), a β-mannosidase experimentally characterized by X-ray crystallography, contained the following conserved domains: “Glyco_hydro_2”; "Mannosidase_ig" and Ig_mannosidase. CB10-00347, which, in turn, were grouped with the sequences of *Bacteroides xylanisolvens* (D4VPT7) and *B*. *vulgatus* (A0A0P0L162) in clade **E**. Unfortunately, both sequences from the Brenda Database were not previously characterized, and the closest characterised sequences in the NCBI database were for β-mannosidase from eukaryotes (Q5H7P5.4 and Q75W54.3). From the point of view of functional domains, the sequences closest to the putative β-mannosidase CB10-00347 presented the following respective sets of conserved domains: 4VPT7, F5/8 type C domain protein and Beta-mannosidase; A0A0P0L162, Glyco_hydro_2 e Glyco_hydro_2_C.

### CB10 growth pattern against different carbon sources

**Fig 3:**
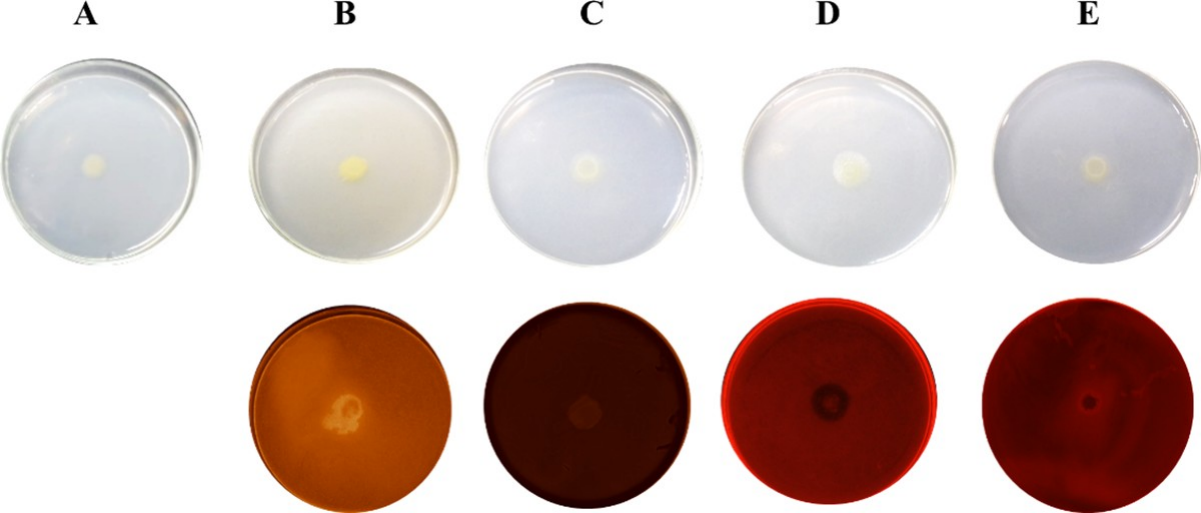
BHB agar medium plates supplemented with different substrates (10.0 g L^−1^), resulting from cultivation after 96 hours, respectively, (A) Glucose, (B) Galactomannan (Gum Locust bean from *Ceratonia siliqua*), (C) Carboxymethylcellulose (CMC), (D) *Aloe vera* and (E) Starch. The lower plates depict the B-E treatments stained with Congo Red (1%).

We evaluated the colony formation capacity of *Chitinophaga* sp. CB10 against five substrates of different structural complexities: Glucose; Galactomannan from the Gum Locust bean; Carboxymethylcellulose (CMC) Pulp extracted from *Aloe vera* leaves, and Starch (Fig 3**)**. After cultivation, the plates were stained with Congo Red to highlight the outline of the colony and possible halos. A greater development of colonies is observed on more complex substrates in the respective decreasing order of larger diameters: *Aloe vera* > Galactomannan > CMC (Fig 3B–3D). As can be seen in the polysaccharide plates stained with Congo red (Fig 3, bottom), the consumption of the polysaccharide can be observed on the border regions of the colony, marked by lighter regions than the surroundings, without great signs of dispersion across the plate.

**Fig 4:**
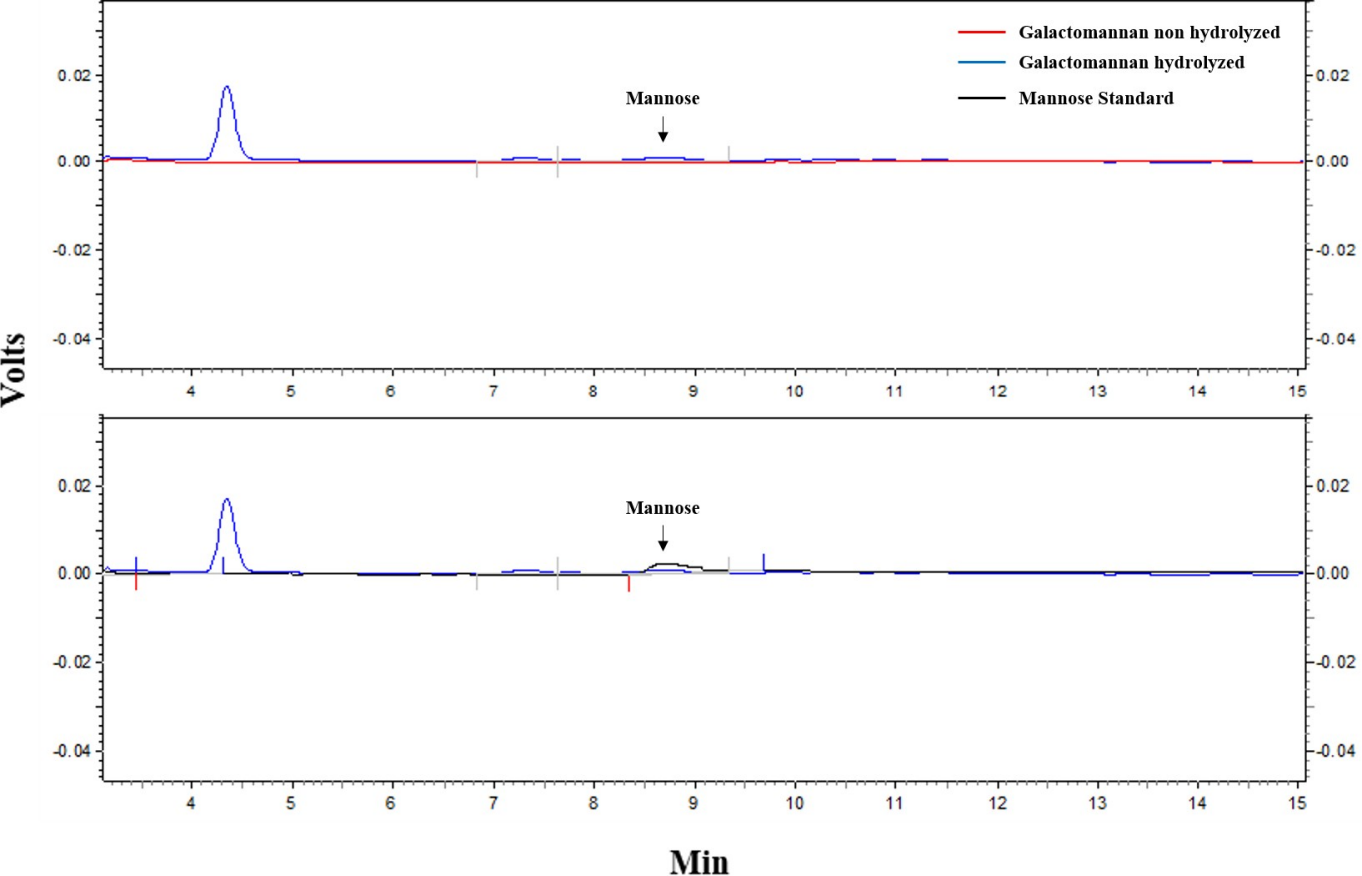
Chromatograms of the sugar profile of the culture medium with galactomannan grown with CB10 were detected by HPLC-RID, demonstrating the degradation of the polysaccharide generating mannose (Blue), as was evinced by the comparison of the overlapping of the pure solution pattern that demonstrates the location of the peak mannose (black), which is absent in the control sample (culture medium without cultivation, red).

In addition to static cultivation in a solid medium, cultures were made under agitation in a liquid medium, aiming at the quantification of the sugars released by hydrolysis by HPLC and DNS techniques, for natural polysaccharide substrates. The evaluation of the acetomannan and galactomannan polysaccharides [50]—respectively, the derivatives of *Aloe vera* and Locust bean (*Ceratonia siliqua*)—in addition to the sugarcane bagasse, which is the biomass from where the bacterium *Chitinophaga* sp. CB10 was isolated, whose hemicellulose is composed predominantly of L-arabino-(4-O-methyl-D-glucurono)-D-xylan, containing small traces of D-mannose [16], was carried out. The liquid culture under agitation might provide a better assimilation of nutrients, allowing the interaction of the particles in solution with bacterial cells, while leading to the dispersion and dilution of toxins produced by bacterial metabolism, which may not occur very well in a solid culture. CB10 was able to vigorously develop and multiply on these substrates when using them as the only carbon source in the BHB medium, which indicates the versatility of its metabolism. However, the oligosaccharides released in the culture medium were consumed almost entirely in their development, so that spare residues were susceptible to detection by HPLC-RID only for the substrate galactomannan, as can be seen in Fig 4, which shows the chromatograms of the sugar profile of the culture medium with galactomannan after bacterial cultivation (in blue) overlapping the treatment control (in red) and the mannose pattern (in black).

In the chromatographic curve corresponding to the bacterial culture supernatant (Fig 4, blue), the presence of two peaks is observed, one referring to mannose (retention in 8.7 min) and the other corresponding to galactose (retention of 7.3 min), in addition to the largest peak, which apparently is an artefact of the chromatographic run, corresponding to the solvent of the mobile phase (4.3 min). The presence of these sugars, absent in the control, provides evidence of the metabolic activity of CB10 on the galactomannan substrate, since the conversion of galactomannan to monosaccharides D-mannose and D-galactose requires the action of at least two enzymes, β-mannosidase and α-galactosidase (although other enzymes are needed for this to happen effectively). Quantification of the samples’ carbohydrates was performed by external calibration in the dynamic working range of 12 to 100 mMol and allowed the detection of 12.34 mMol of mannose in the CB10 cultures in BHB supplemented with galactomannan. The DNS analysis corroborated the data obtained from HPLC-RID, so that the cultures with BAG and ALO did not allow the detection of sugars released by hydrolysis, even when the samples were concentrated 5X. For the galactomannan culture, the DNS test allowed the estimate of the amount of reducing sugars released at 2.32 μg/mL.

### Bagasse versus glucose differential expression of mannosidases

**Fig 5:**
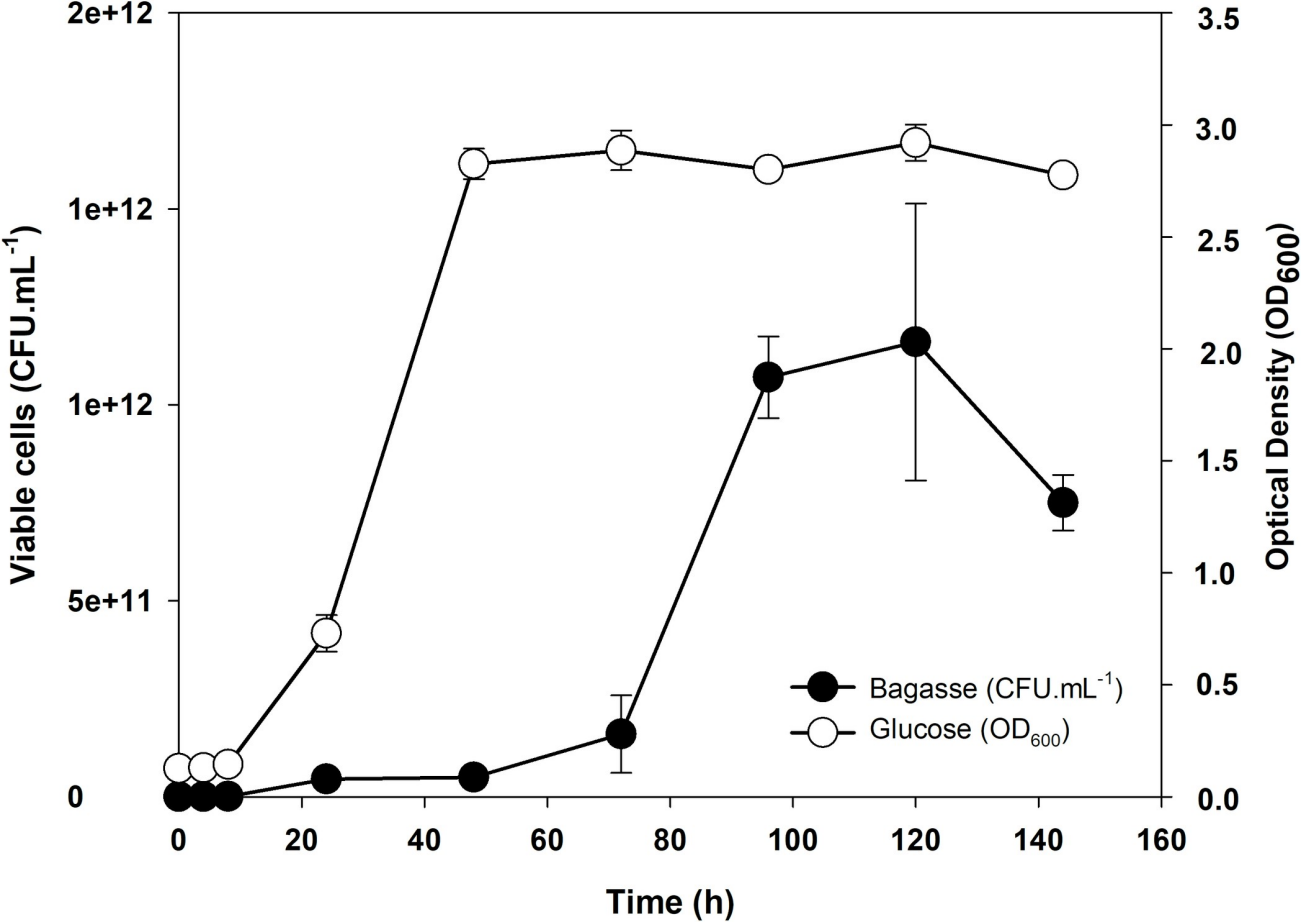
Bacterial growth curves of *Chitinophaga* sp. (CB10), in a solid BHB culture medium with sugarcane bagasse by Colony Counting Unit (CFU) and in a liquid BHB culture medium added with glucose by reading Optical Density (DO_600_).

A real time expression analysis for two β-mannosidase GH2, CB10-153.446 and CB10-00347 in Bagasse was performed. Initially, the growth of CB10 against bagasse (BAG) and glucose (GLU) as carbon sources were analyzed, aiming to identify the bacterial development profile in each of the cultures. Fig 5 shows the development curves of the CB10 bacterium in the BHB minimal culture medium for the BAG and GLU treatments, by the respective Colony Forming Units (CFU) counting, and by optical density (OD_600_) techniques. The preparatory adaptation phase of the cultures (Lag) showed a slight initial differentiation and, for up to eight hours, both treatments remained with apparently very low cell multiplication, below the detection limit of the techniques used. The adaptation phase was shorter for the GLU treatment, extending for up to 24 hours, when the cultivation shifted to exponential growth. For the BAG treatment, this adaptation phase was longer, going through a period of slow multiplication for up to 72 hours, when the log phase actually started. Considering the distinct nature of the analysed carbon sources, this difference in the adaptation phases is fully comprehensible, since glucose is a simple sugar, readily absorbable by most bacteria and processed in its metabolism; while lignocellulosic biomass is a source of extremely complex carbon, requiring the induced expression of several enzymes and demanding a longer adaptation phase for the culture. Both curves declined in 144 hours of cultivation, a fact that can be justified by the scarcity of nutrients as well as by the increase in potentially toxic cell rejects. The bacterial growth pattern of the CB10 bacterium was a guide for choosing the points to be analysed by real-time PCR (RT-qPCR), corresponding to 24 (lag phase), 96 (stationary phase) and 144 hours (decline phase).

**Fig 6:**
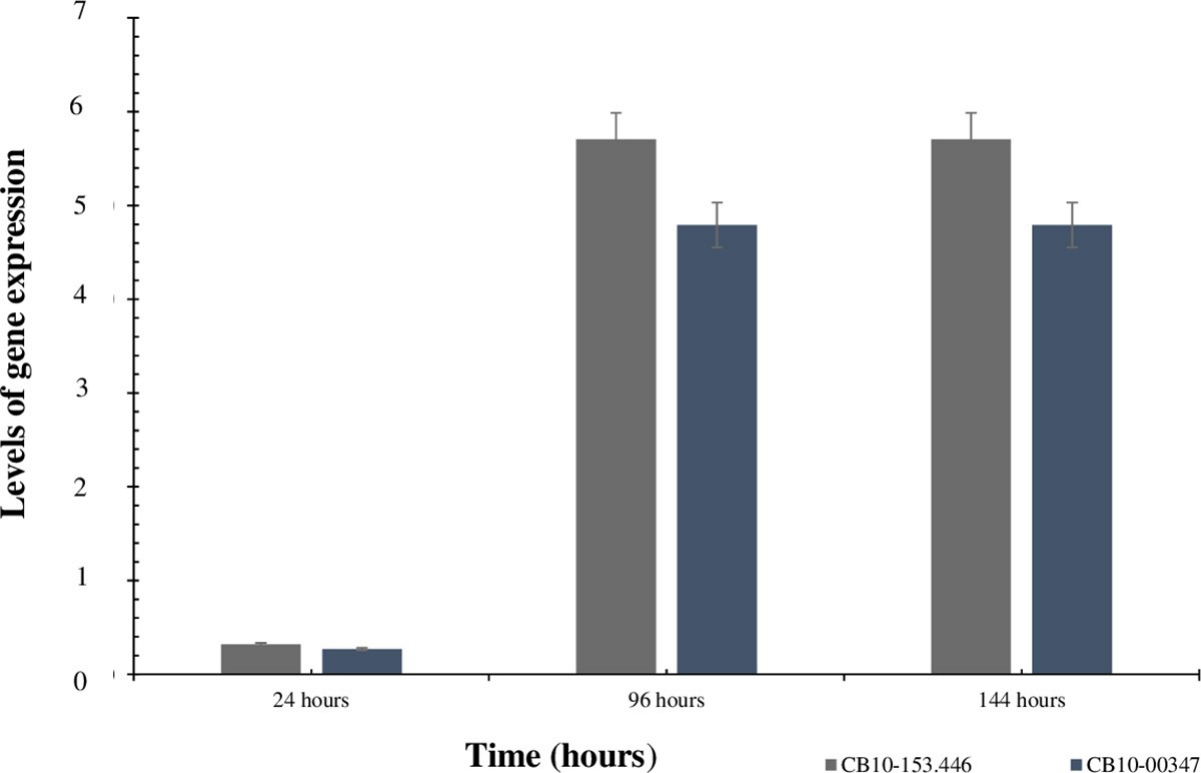
Level of expression of the β-mannosidases genes, respectively associated (CB10-00347) and not associated (CB10-153.446) to PUL-like clusters, calculated using the 2^-ΔΔCT^ method for *Chitinophaga* sp. (CB10) in a liquid BHB culture medium with sugarcane bagasse in relation to endogenous control (SigmaA) and negative enzyme induction (glucose), in the periods of 24; 96 and 144 hours.

The results of increasing levels of gene expression in samples of the CB10 bacterium cultivated in the sugarcane bagasse at 24, 96 and 144 hours in relation to the same periods of glucose cultivation (negative control of expression induction) are illustrated in Fig 6. It is observed that in the period of 24 hours the expression levels of both enzymes remained at baseline and equal levels of expression, both being very low when compared to those obtained in the periods of 96 and 144 hours. After 96 hours, a condition of induction of gene expression that was different from the non-induced control (glucose) and the level of expression constituting endogenous control (SigmaA) was observed, with the respective levels of 2^-ΔΔCT^ values for CB10-00347 and CB10-153.446, showing an increase in expression of 4.8x and 5.6x, being, therefore, lower for β-mannosidase located within the PUL-like pathway. Curiously, both the 96-hour and the 144-hour periods showed a high stability of the level of specific gene expression for each gene over time, and the respective values remained practically the same for both times (S1 and S2 Figs)

## Discussion

Bacteria from the phylum Bacteroidetes are complex carbohydrate degrader agents that collectively encode a wide diversity of GHs in their genomes, including both conventional secreted enzymes and special enzymes located in gene clusters named “Polysaccharide utilization loci”, or PUL [51, 52]. PULs have quite intricate mechanisms, with the organization of some carbohydrases fixed to the plasmatic membrane or confined in the periplasmic space; in addition to the presence of specific channels for the entry of oligosaccharides into cells (SusC/TonB) and polysaccharide-binding proteins (SusD), this latter acting to connect bacteria to the carbon source. Each PUL encodes specific proteins involved in the recognition, binding and hydrolysis of a particular type of carbohydrate [53]. One example of this could be observed in a recent study in which the grown using mannan conjugates induced the expression of carbohydrate active hydrolases, likely by the action of PULs, in *Chitinophaga pinensis* [8].

From the point of view of biological enzyme arsenal, the Bacteroidetes bacterium subject of this study, *Chitinophaga* sp. CB10, is extremely promising, holding a variety of CAZymes (350 predicted domains), and studies still in progress have strongly indicated that CB10 is a new specie of the genus *Chitinophaga*. Most of these CAZymes correspond to GHs (202), of which 27% were located in about thirty PUL-like pathways identified along the full genome, including one GH2 β-Mannosidases PUL-like associated (CB10-00347, Fig 1, S3 File) and one GH2 β-Mannosidases without association to any gene cluster (CB10-153.446). The homology analyses of these enzymes by global alignment (BlastP /NCBI) against the “Non-redundant UniProtKB/SwissProt” database indicates a high degree of novelty once the sequences shared between 28–43% with previously characterized enzymes. In phenetic dendrogram results, the sequence of CB10-153.446 grouped with sequence Q8AAK6, a GH2 β-mannosidase from *Bacteroides thetaiotaomicron*, named BtMan2. *B*. *thetaiotaomicron* belongs to the natural human gut microbiota and has an important role in human health balance. BtMan2 showed a pH optimum of 5.6 and thermo tolerance up to 58°C, which could be especially useful for industrial processes. Interestingly, the enzyme was shown to be completely resistant to proteolytic attacks, indicating it is a suitable enzyme to act as an extracellular hydrolase. This is very compatible with its environment, since the gut is rich with proteolytic enzyme secretion [54]. By homology inference, these results agree with the hypothesis that CB10-153.446 is an extracellular enzyme, which is reinforced by annotations without association to any PUL cluster.

The GH2 β-Mannosidase putative sequence CB10-00347 grouped with still non- characterized sequences, thus more studies are needed to understand its role in the *Chitinophaga* sp. CB10 metabolism. Moreover, as can be seen in the PUL annotation details (S3 File), the CB10-00347 was predicted by prokka as an “exo-beta-D-glucosaminidase precursor”, probably due to one of its three domains being recognized on the Pfam Database (http://pfam.xfam.org/): Glyco_hydro_2, Glyco_hydro_2C domains and Exo-beta-D-glucosaminidase. According to Côté and co-works [55], several exo-beta-D-glucosaminidase (GlcNase) have been annotated in databases as putative β-mannosidases, which hinders the prediction by homology, frequently demanding experimental steps for the correct classification [55]. Furthermore, CB10-00347 had a substantial expression level in bagasse, that does not have any GlcNase substrates (i.e. chitosan hydrolysates, often from fungi sources), which supports the hypothesis that it is a new β-mannosidases.

Bacteria of the genus *Chitinophaga* sp. have a wide spectrum of assimilable carbon sources for their development, whether these sources are formed by a simple structure such as dextrin, maltose, trehalose, cellobiose, gentiobiose, sucrose, turanose, stachyose, lactose, melibiose, methyl b-D-glucoside, D-salicin, N-acetyl-D-glucosamine, N-acetyl-b-D-mannosamine, N-acetyl-D-galactosamine, N-acetyl-neura-minic acid, a-D-glucose, D-mannose, D-fructose, D-galactose, D-fucose, L-rhamnose, D-sorbitol, D-mannitol, among many others [56] or by several types of complex polysaccharides like glucomannan, galactomannan, lignocellulosic biomass of softwood [2, 8], dead fungi biomass [50] or grass biomass such as the sugarcane bagasse [9]. Regarding the ability in grown using different substrates, our results showed that in a solid medium, CB10 was able to develop in different sources of carbohydrates, such as carboxymethyl cellulose, starch, *Aloe vera* gum and galactomannan, revealing a great potential of nutritional plasticity, which agrees with the great variety of CAZymes present in this organism.

Congo Red is a water-soluble dye, able to stain cellulose, amyloid fibrils, agricultural starch products and others polysaccharides, which makes it a good test tool to indicate polysaccharide consumption by bacterial growth in a culture media [57]. Thus, we used this dye to stain the plates and highlight the outline of the colony and possible halos. In common with observations from previous works, the development of the colony in starch was inferior to other polysaccharides [10, 58]; thus its diameter profile is similar to what is found for glucose, which also did not stimulate the increase in colony cell mass. Apparently, in the cultivation conditions evaluated for the substrate in a solid medium, the most complex substrates (Mannans present in *Aloe vera* and Gum Locust bean plant extracts) could offer more nutrients than the simplest sources (Glucose, CMC and Starch). The latter group essentially offers only glucose as the final carbohydrate for assimilation, which seems to have given complex substrates greater advantages over simple carbon sources under static growth conditions (Fig 3). Carrysin is the predominant linear acetomannan in the gum of the plant extract of *Aloe vera*. It has a backbone of β-(1→4)-linked D-mannosyl residues, with C-2 or C-3 acetylated and some side chains of mainly galactose attached to C-6 [12]. Locust bean gum is formed by some residual impurities (1.2% of minerals and plant extracts) and mostly by a neutral galactomannan polysaccharide composed of mannose units spaced every four units by side branches of galactose units [12] (Sigma Aldrich^®^). Galactomannan is the target of research in several biotechnological fields, mainly in the food industry, as a thickener, being a food additive with low toxicity and low cost availability [59].

In Bacteroidetes, PULs are identified as the main means by which the production of CAZymes is regulated in response to the availability of carbohydrates [53]. In addition, the literature has recently reported the importance of regulating systems similar to PULs in other phyla, such as the mechanism related to the use of mannan in *Bacillus* sp. in Firmicutes [60]. Many of the PUL pathway enzymes are responsible for the natural deconstruction of biomass in order to acquire nutrition from decomposing plant material. These important enzymes present in microbial genomes are considered a rich source of industrially useful enzymes [19, 53]. However, though the anchorage of hydrolytic enzymes from PULs on fixed structures into the bacterial membrane and cell wall facilitates the cellular absorption of hydrolysed monosaccharides, it prevents the dispersion of the enzymes in the extracellular medium. This characteristic may relatively lead to the lack of dense halos in solid culture plates since there is no dispersion of most hydrolases in the system outside the limits of the colony’s border. As can be seen in the polysaccharide plates stained with Congo red (Fig 3, bottom), the consumption of the polysaccharide can be observed in the border regions of the colony, marked by lighter regions than the surroundings, without great signs of dispersion across the plate. This may indicate that in the 96-hour time period, for the static cultivation of the tested substrates, apparently no enzymes were produced that were excreted in free systems by the bacteria, with only enzymes in some way associated with the bacterial wall, either by transmembrane or potentially associated with a PUL-like system.

In agitation culture (liquid), the organism also presented a vigorously grown, including with treatment in which only natural substrates were used as carbon and nutrient sources (sugarcane bagasse, *Aloe vera* and galactomannan gums). In spite of this, among those natural substrates, only the hydrolysis of galactomann allowed the detection of oligosaccharides in the culture medium, which indicates that the released sugars were used almost entirely in the development of the bacterial culture.

According to Cano and co-works [61] the oligosaccharides are often arousing intermediate compounds resulting from side processes from many biorefinery approaches for biomass. They have many applications in biotechnology for pharmaceutical, nutraceutical, cosmetic and food industries. Among plant biomasses, food crops are very promising to facilitate obtaining oligosaccharides by biorefinery, mainly cellooligosaccharides (COS), pectooligosaccharides (POS), xylooligosaccharides (XOS) and others, considered less abundant, such as oligomers containing mannose, arabinose, galactose and several sugar acids [61]. Considering that more abundant oligosaccharides are easily obtained from different sources, as, for example, what occurs with the autohydrolysis process of different biomasses (corncob, rice husks, barley husks, vine shoots, chestnut shells and peanut shells, among others) that result in XOS production [61], new approaches need be developed to expand the availability of rare oligomers. Between these new approaches, we may cite the search for new enzymes as well as the search for non-obvious biomasses, even those composed by less targeted oligomer content, once new material sources have a greater chance of success in leading new chemical scaffolds.

Frequently, the low mannan content (0.2%) in the sugarcane bagasse does not stand out in the bioenergetics and biorefinery studies of this biomass, since the focus of these studies is the xylans from hemicellulose and fermentable sugars derived from cellulose [16]. Nevertheless, oligosaccharides of different complexities can be obtained through biomass biorefineries, which may have unique properties [27]. As previously reported above, CB10 was isolated from a lignocellulose-degrading bacterial consortium obtained from a pile of sugarcane bagasse, and its genome has impressive plasticity for carbohydrate degradation over its two hundred GHs, among other domains of CAZymes. The functionality of these GHs is made evident in the ability of this organism to use carbon sources of different degrees of complexity (Figs 3 and 4), and these enzymes seem to be part of the enzyme complex responsible for acting on the acetomannan and galactomannan metabolism, allowing the microorganism to survive using these mannan-rich carbon sources.

Once the existence of functional enzymes for mannanases in isolated CB10 is proven, and considering that recent studies have reported mechanisms of expression of mannanases in *Chitinophaga pinensis* for rich gluco- and galactomannan compounds [8, 50], the aim of this study was to investigate whether the two β-mannosidases annoted in the genome would be expressed in the sugarcane bagasse, where the portions of polymer containing mannose are apparently negligible, while the abundance of other polymers is evident [16]. The results showed considerable gene expression after 96 h of cultivation in the sugarcane bagasse as a carbon source: around five times more than what could be observed for the negative expression control (glucose), remaining stable for 144 h. Thus, sugarcane bagasse could lead to expression of mannanases, even though other more abundant carbon backbones were present. These indicated that further studies detailing the chemical composition for diverse fractions during the degrading time course could lead to the discovery of new oligo intermediates. Next, the rates of the new compound would be incremented by using purified enzyme cocktails, as well by using engineered organisms to prioritize rare biochemical routes instead of the more abundant ones.

## Conclusions

Our results revealed that the CB10 bacterium is able to activate its metabolism related to mannan degradation even in conditions where the amount of the compound is extremely low (0.2% in the bagasse), expressing both an enzyme related to the PUL mechanism and one apparently of independent performance, the expression of which is about 14% higher. The results showed a considerable and stable gene expression for the period of 96 h, and these levels remained practically the same for the phase of decline (144 h). The high number of CAZyme domains identified in CB10 apparently gives the organism plasticity, allowing it to develop in all evaluated carbon sources. The ability to use different plant biomasses as a carbon source also indicates a high potential for the production of enzymes for biotechnological applications in several fields, such as the food and pharmaceutical industries, while the literature indicates that the hydrolysis of bagasse biomass and *Aloe vera* and locust bean gums are widely promising for the production of low-toxicity functional carbohydrates.

## Supporting information

S1 File Sequences of β-mannosidases used in this study, annotated by homology from draft genome CB10 (NCBI ID number: MLAV00000000). (DOCX)

S2 File Multiple alignment of the GH2 β-mannosidases protein sequences (CAZy and BRENDA databases) with sequences of this study (yellow), performed by T-coffee. (DOCX)

S3 File Details of annotated genes clustered in “PULs-like” associated with CAZyme GH2 β-mannosidase CB10-00347. (XLSX)

S1 Fig The RNA integrity, assessed using the device Agilent 2100 Bioanalyzer. (TIF)

S2 Fig Amplification curves RT-qPCR. (TIF)

S1 Table Rt-qPCR raw data. (DOCX)
