# Supplementary material for: Bagasse minority pathway expression: Real time study of GH2 β-mannosidases from bacteroidetes
Source: PLoS One. 2021 Mar 17;16(3):e0247822. doi: 10.1371/journal.pone.0247822 (PMC7968711; doi:10.1371/journal.pone.0247822)
Supplement: S1 File — (DOCX) [file pone.0247822.s001.docx]

**S1 File. Sequences of β-Mannosidases used in this study, annotated by homology from Draft genome CB10 (NCBI ID Number: MLAV00000000)**

>CB10-153.446

TTTGCTGGTGATCATGAATATCAAGCTGACGGGTCGCTTTTTTTTTTATCCTTTTTTACTAAATCGATTTACTCACAGGTAGACACGCTGAAGAGTGGATGGGAATTTGGTCGCCCGGATGAAAGGATCTGGCGGAGCGCTACCGTTCCGGGAACGGTACATACCGACCTGCTGGCGCATCACCTCATCCCCGACCCGTTCAAAGGCATGAACGAAAAAGCCGTGCAATGGGTAGATAAAAAGGATTGGTGGTACCGCTGCACCTTCAACGTTACGGAAGCGCAACTGAAGCAGGACGTAGTGGAACTGGAGTTCCTCGGCCTCGATACCTACGCGGAAGTATCCGTCAATAACCGGGATGTGCTGGTAACCAACAACATGTTCCGCGCATGGTCCGCCGATGTGAAGCGGCACCTGAAACCAGGAAAAAACACCCTGCTGATCAAGTTTGAAAGTCCGATTAAGCACGATATGCCACGCTTCCTCCGGGATAGCGTGATTTACCCTGCCGGCAACGATGCCAGCGATATCCCCCTCAGCGTGTACGCCCGCAAAGCGCCCTACCACTATGGCTGGGACTGGGGCCCGCGCCTCGTTACCAGCGGCGTATGGCGCCCGGTGATCCTCAAAGCATGGAGCGCCGCTACCATCCGGAACGTAAGCTATACCACCGCCCTCGAAGGCAAAAAAGCGCGGGTAAATGTGAAAGTGCAACTTACGGTAACCCGCCCGGGCACGTACTTGCTGACTCCTGCGCTGACTCTCGGCAACAGTTCCGCGATAGCGAACCGCCCCGGTAAAGCGTTTACCCTCCCTGAAGGCGATACCACCTGCGAACTGAGCTACGAGGTGAACTCGCCGCAACGCTGGTGGCCCAACGGCATGGGCGCGCAACCGTTATACCAGGCCACCGTGTACCTTACCACAGCAGAGAAAACAACGCTTGCGCAGCTGAGTAAGAAAATCGGATTCCGTACCATCGAAGTGGAAAATAAACCGGATGCACAAGGCGAATCATTTTTCGTGAAAGTTAATGGCCGCCCGGTTTTCATGAAGGGCTCCAATTACATCCCGCAGGATAATTTCCTGCCCCGTGTAACAAAAGAAAAATACCGCCGGCTGTTCGACGATATGCAGGAAAGCCACTTTAACATGGTGCGCGTGTGGGGCGGCGGCATATACGAAAATGATGAGTTCTACGACCTGGCGGATGAAAAGGGTATCCTCGTCTGGCAGGATTTCATGTATGCGTGTACGTTATACCCGGGCGACAGAACCTTCCTCGAAAACGCCCGTATGGAAGCCATCTACAACATCAATCGCTTAAAAGATCACGCCTCCCTGGCACTCTGGTGCGGCAACAACGAAATTGCCGTGGCGATCAAAAACTGGGGCTGGCAAGACGGTTACGCCTATACGAATTTGCAGTATGAAAGCATGCAGCGTGCGTATGATAAACTGTTTAAGGAGATATTACCCAACGCGGTGAAGGACCATGATCCCGGCCGTTTCTATTTCCACTCTTCTCCCATCAGCAACTGGGGAAAGCCGGAAGATTTCACCAGGGGAGATAACCACTACTGGGGCATCTGGCATGGTATGGAATGGTTTGAAGCGTTCAACACGCACATTCCCCGCTTCATGAGTGAATACGGCTTCCAGTCGTTCCCCGGCATGGCAACCATCGATAGCTTTGCCACCAAAGACGACTATCACATTTTCTCCAACGTGATGCAATCCCACCAAAAGAGCCCTGCTAAAGGAAATACGGCGATCAAAATTTACATGGACCACTACTACAACACACCGGTGGATTTTCCTGCGTTCGTGTACCTTAGCCAGGTATTGCAGGCGGAAGGTATGAAAGTGGCCATAGAAGCGCATCGCCGCAATATGCCTTACTGCATGGGCACCCTCTACTGGCAGCTCAATGATTGCTGGCCAGGCCCGTCCTGGTCCGGAAGGGATTACTATGGAAGATGGAAGGCGCTCCAATACTACGCCAAACGGGCGTTCGAACCAGTGATCACGTCGACAGTGGAGGAAAATGGGCAGTTAAAGACTTTCGTGATCACGGCTGAACATGGAAATAACGCTAAGTGGGATTTGGAGCTAAAACTGGTACTGACTGACCTGCAGGGCCAAATTATCTCCCTAAAAAAAATACCCGCACGTAAGTATGCCGATGGGGCCAGCACCCTTGTAAACACTGTAGCAATCAATGATTTATTGCAAGGCCGGCCCCGTAATGAGGTAATATTCTATACTGAATTGGTAAATAACAATAATGTTCTGAATCCTAATCTCTATTATTTTGCAGCAGCGAAAGAGATCGCGTTACAGGAACCCGGCATATCCTTTACCTGCAAGGCCGCAGGCGATGGAGTGGATGTAACCGTTACAACAAAACAGCTTGCGAGAAATATTTACCTGGGACTTTCAACAGCAATTGAAGGAGAACATTTTGAAGATAATTTCTTCGACCTGCTGCCCGGCATGCAAAGGACCGTGCATCTGCGTACCGGCCGCACCGCGGCAGCCATACAACAACAGCTGAAAATAACTTCGTTGGTTGACACTTATAAAAAATAA

>CB10-00347

ATGACGCAACGACTCATCTTATCCTCACTCATACTCCTCGCCGGCGCCCACCTCCGCGCCCAGGAACAATATGAGCTGAACAGCGACTGGAAATCAATGCCAATAAAGGAAGTAAAGCTCGACGGAACCGCCATCTCCCAACCCGGGCAGCAACTCCGCAACTGGCAACCCGCCATCGTGCCGGGCACCGTCCTCACCACCATGCTGCACAACAAACAAGTACCGGACCCTTTCTTCGGCATGAACAACAACCGTATCCCGGATATCTATGCCACCGGGCCGGAATACTATACCCGCTGGTTCGTTAAGGACTTCACCGAACCCGCCCCCGCTGCGGACGGGCAGGTATGGCTGCACCTGCGCGGCGTGAATGACGGCTGCGATATCTACCTGAACGGAAAAAAAGTAAACCCCGCCACGCACCACGGCGCTTACCTGCGCCAAACCTATAACATCACTTCCTTCCTCGCCAGGGATGGTAAAAACCGCCTGGCAGTCATCGTATATCCGCCGGCGATACCCGGGAACCCCAACGGCGGCCAGGGAGGCGATGGCGCCATCGCGCGCAACGTAGGCCCGCAGTACACGGCAGGGTGGGACTGGATCCAACCCATGCGGGACCGCAATACCGGCATCTGGGATAAAGTAACCATCGAAAAAACCGGCGCCGTACGTATCAAGGACCCGCACGTCATCACCCTCGTGCCAGGCATACGCCAGCCGGAAGGCCCTCAGGCGCCCGCCACTATCAAAGTATCCGCCACGCTGGAAAACGCCGCTGCTACCGCTGTATCCGGCGTGCTTAAATATGTATTGAATGGTAAATCGATTCAGCAACAGGTAACCCTTGCCCCTAAAGGTATTACGACGGTCGCCCTGCCGGACCTGCAGCTGGACAACCCCCGGCTCTGGTGGCCCGCCGGCTACGGCCCCCAGCACCTCTACCAGCTGCCGCTGGAATTCACCGCCAACGGCAAAGTGAGCGACCGGGAAAACGTAGAGGTGGGCGTACGGGAAATACAAACAGCCTGGAACACCCACACCCGCAGCAGGGAAATCCGCGTAAACGGACAACGCATCTTTATCAAAGGCGGCAACTGGATCACCTCCGACGCTATGTTCCGCTTCAGCAAAGAACGCTACGACGCGGAAGTGCGCTTCCACCGCGATATGAACCTCAACCTCATCCGCATCTGGGGCGGCAGCCTCACGGAAAGGCCCGAGTTCTTCGAAGCCTGCGATAAATACGGCCTGCTGGTGTTCCAGGACTTCTGGATGTCCGGCGACTGCAACGGCCGCTGGCTCGACCCTAAAAAGAAGGACGACCAGTGGACCCGCCGCCAGTATCCCAACGACCACGGCCTGTTTATCCGCAGCGTGGCGGACCAGGTAAAAATGCTGCGCAACTACCCATCCCTCGCAATCTGGTGCGGCGGTAATGAAATCACCCCGCCGCAGGACATCCTCGCCGCCATGAAGGATAGCATCCTCCCGGTGCTGGATGGGACCCGCTACTTCTTCGACTATTCCAATACCGACAGCATGTCCTACAACTTCATCGGCGGCAATGGGGACGGGCCTTACGGCATCCAGCCAATAGAGCGCTTTTGGGCCCACCGCACCTTCCCGTTCAACTCGGAAGTAGGCTCCGTGGGCGTGGGGGATATCGCCAGCCTCAAAAGATTCCTCCCGGCCGAAAACCTTGTAGCGCCTGACTTTGCAGCCAATAAAGTGGACAGCGTATGGGATTATCACAAATACATCTCCTATGACAACCACCTGGCGCCATATGGCCCCGCGAAAAACCTGGAAGACTGGGCCAACAAAGCGCAACTGGTCAATTACGACCAGTACCGCGCCCTGATGGAAGGCTTCAGCGCGCATATGTGGGACTGGTACACCGGTACCATCATCTGGAAAACGCAGAATCCATGGACGGCCTTACGGGGACAGATGTACGATTATTACCTCGATCCCAACGCCTGCCTGTATGGCCTCCGCAGCGGCAGCAAGCCCTTGCACGCCATGTACGACCCGGTGAATAAAATCGTCATGGTGGCGAACAATACCTTCCAATACCACCGCGATCTCATGCTGGATGTACACGCATACGATATGGCCGGCAAACAATATCCCATAGATCAATTATTCGTGGAGGCAACCCCGTCGAATGTCCAACGCCATATTGAATTGAAAACAAAGCTGGACGCACTGAAAGAGAAAAAGGGCCTCTTCCTTTCCCTGCGGCTCCGTAATCTCCGCAAGGAAATAATAGATGATAATTTCTATTGGTTGCCTGCGGCAGATGGTAACTACAGCGGCTTACAGGAGCTGCGGCCGGTCAAACCAACGATCACGGCTATACGCCGAAATCAGGAAGTACTCGTTACCATCCATAACCCTGCAAACGGGCCGGTCGCCTTTTTCAACCGGGTTTCCCTGCTGGATGCGAAAACTAAAGAAAGAATATTGCCGGTATTTTACTCCGATAATTACGTGTCGGTATTACCGGGTGAAGAGCGGCAGATCGTGATCAGCGGCGACAGGGCAGCCGCCTCAGGCAATGCGCTGGTAGAAGTGTACGGACAAAACGTTGCCCCGCAGCAGGTGAATATTCAATAA

> CB10_04158

ATGTCAATGCGCCAACTCAAAATCACTAAATCCATTACCAACAGGGAATCACAATCCCTGGAAAAGTATCTGCAGGAGATTGGGAAAGTGGATTTAATTACGCCGGAGGAAGAGGTAAACCTCGCCATCCGCATCAAGCAGGGCGATCAGAGAGCGTTGGAAAAGCTTACCAAAGCCAACCTGCGTTTCGTGGTTTCCGTTGCCAAACAGTATCAGAATCAGGGCCTGTCGCTCAGCGATCTTATCAATGAAGGCAACCTGGGGTTAATTAAAGCTGCTCAACGTTTTGATGAAACGCGCGGTTTTAAATTCATCTCCTACGCCGTATGGTGGATCCGTCAGTCCATCCTCCAGGCTTTGGCTGAACAGTCCAGGATCGTGCGCCTGCCGCTCAACAAGGTAGGATTGAGCAACAAGATCAGTAAAGCTTACTCCCAGCTGGAACAGGAGTTCGAGCGTGAACCATCTCCGGACGAGCTGGCCACCATTCTCGAAATCAATACAGATGAAGTGGAAGCTACGCTGGGTGTTGCCGCCCGCCACGTGTCGATGGATGCGCCGTTTATCGACGGGGAAGACAACTCCCTGCTGGACGTACTCGAAAATCCGAACGCCGTTAGCGCGGACGAGGAGCTGGATCACCACGATTCCCTCCGCCGCGAGATCGAACGTTCCCTCTCTACCCTTACCGACCGCCAGAAGGACGTCATCATGCTGTACTTCGGCATCGCCGTGGAACACCCGATGTCCCTGGAAGATATAGGAGAGAAATTTGGCCTCACCCGGGAGCGCGTACGCCAGATTAAAGACAAAGCGATCACTAAACTGAGAACCACTTCCCGCAGCAAATTGCTCCGCAATTACCTGGGAAGCTGA
