# Supplementary material for: Bagasse minority pathway expression: Real time study of GH2 β-mannosidases from bacteroidetes
Source: PLoS One. 2021 Mar 17;16(3):e0247822. doi: 10.1371/journal.pone.0247822 (PMC7968711; doi:10.1371/journal.pone.0247822)

**S2 File. Multiple alignment of the GH2 β-Mannosidases protein sequences (CAZy and BRENDA databases) with sequences of this study (yellow), performed by T-Coffee.**
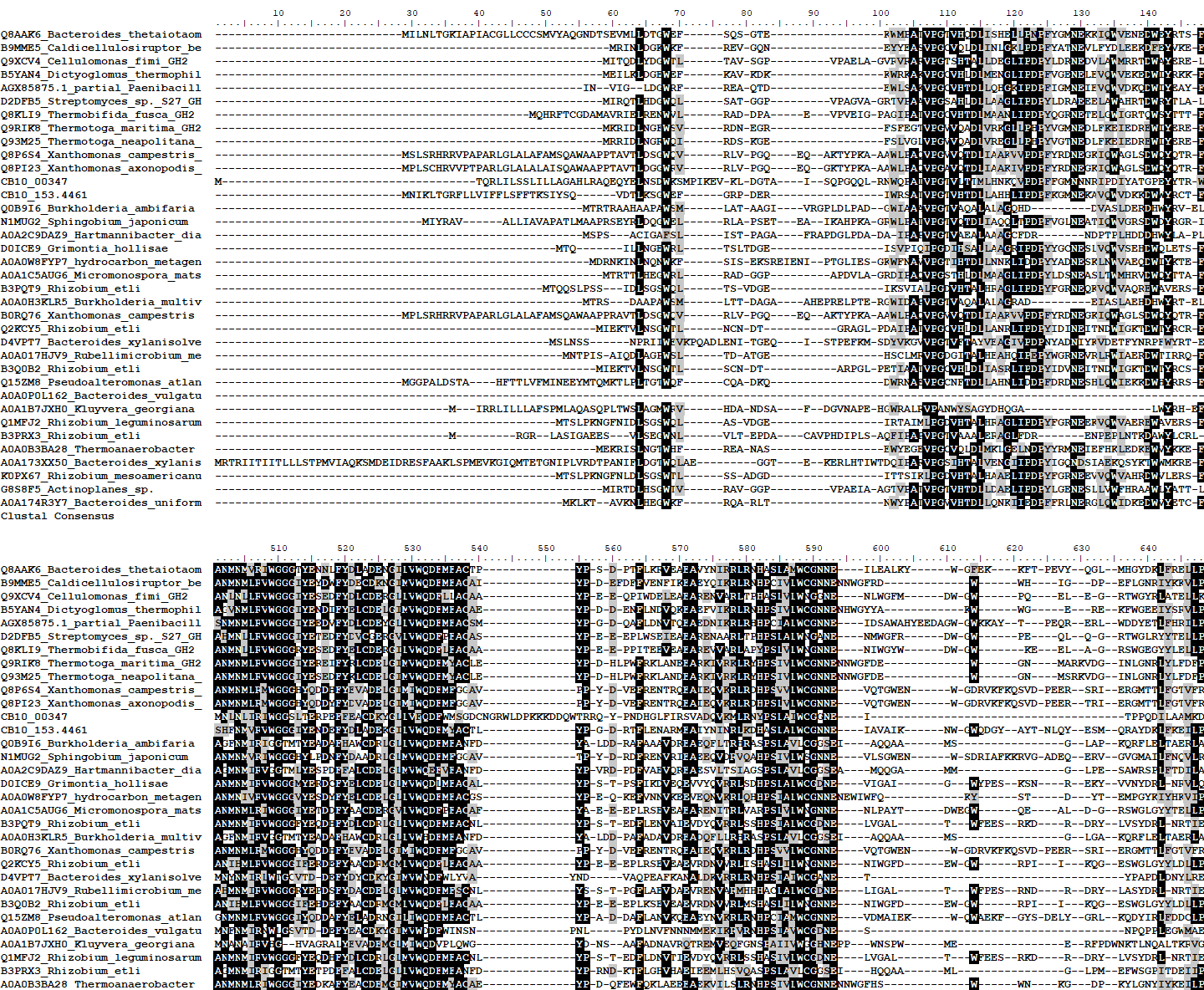


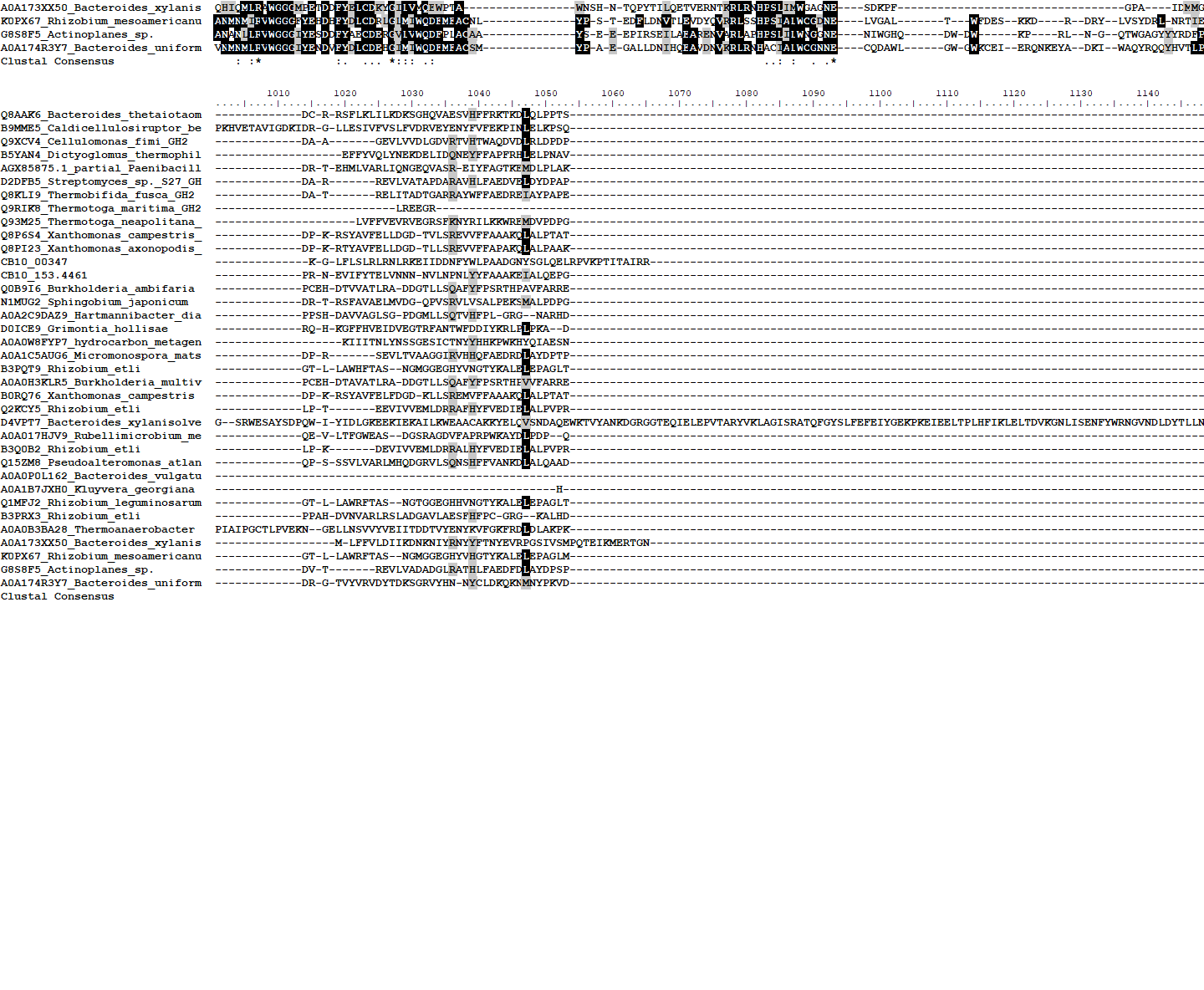

Supplement: S2 File — (DOCX) [file pone.0247822.s002.docx]
