# Supplementary figures and images for: Bagasse minority pathway expression: Real time study of GH2 β-mannosidases from bacteroidetes
Source: PLoS One. 2021 Mar 17;16(3):e0247822. doi: 10.1371/journal.pone.0247822 (PMC7968711; doi:10.1371/journal.pone.0247822)

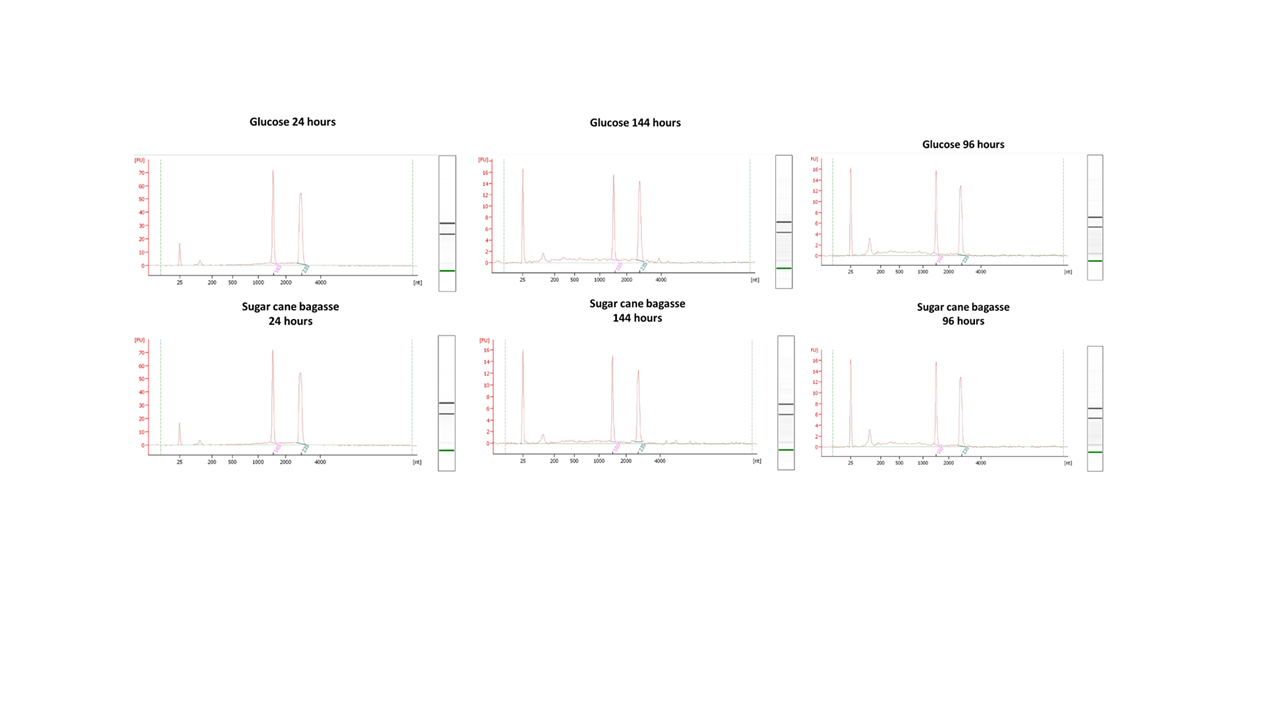

Supplement: S1 Fig — (TIF) [file pone.0247822.s004.tif]

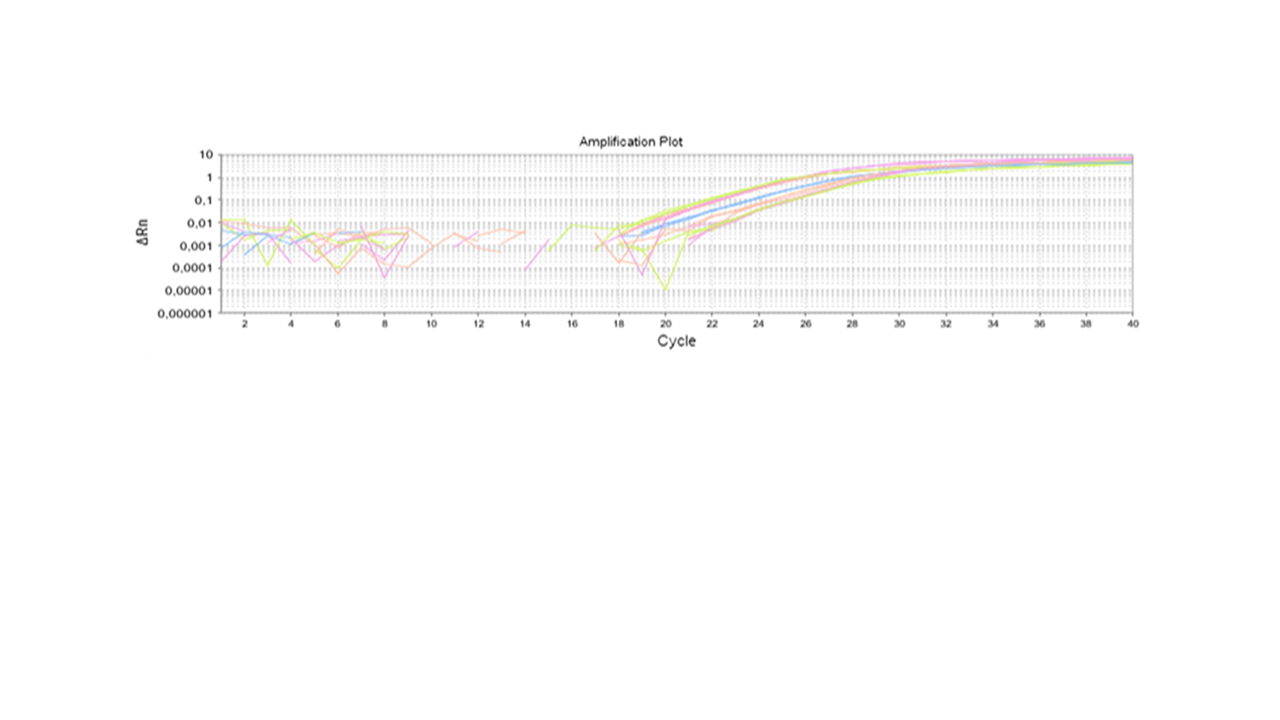

Supplement: S2 Fig — (TIF) [file pone.0247822.s005.tif]
