# Supplementary material for: Bagasse minority pathway expression: Real time study of GH2 β-mannosidases from bacteroidetes
Source: PLoS One. 2021 Mar 17;16(3):e0247822. doi: 10.1371/journal.pone.0247822 (PMC7968711; doi:10.1371/journal.pone.0247822)
Supplement: S1 Table — (DOCX) [file pone.0247822.s006.docx]

**S1 Table. Rt-qPCR raw data**

| **Biological triplicate** | **Sample treatment** | **Name Gene/Time (hours)** | **Ct** | **Average/ΔCt** |  | **Sample treatment** | **Name Gene/Time**  **(hours)** | **Ct** | **Average/ΔCt** |
| --- | --- | --- | --- | --- | --- | --- | --- | --- | --- |
| 1 | Bagasse | CB10_04158 24h | 25.069 | -0.563 |  | Glucose | CB10_04158 24h | 20.479 | -4.544 |
| 2 | Bagasse | CB10_04158 24h | 25.069 | -0.563 |  | Glucose | CB10_04158 24h | 20.479 | -4.544 |
| 3 | Bagasse | CB10_04158 24h | 25.069 | -0.563 |  | Glucose | CB10_04158 24h | 20.479 | -4.544 |
| 1 | Bagasse | CB10_04158 96h | 22.448 | -0.633 |  | Glucose | CB10_04158 96h | 25.916 | -0.452 |
| 2 | Bagasse | CB10_04158 96h | 22.448 | -0.633 |  | Glucose | CB10_04158 96h | 25.916 | -0.452 |
| 3 | Bagasse | CB10_04158 96h | 22.448 | -0.633 |  | Glucose | CB10_04158 96h | 25.916 | -0.452 |
| 1 | Bagasse | CB10_04158 144h | 22.448 | -0.633 |  | Glucose | CB10_04158 144h | 25.916 | -0.452 |
| 2 | Bagasse | CB10_04158 144h | 22.448 | -0.633 |  | Glucose | CB10_04158 144h | 25.916 | -0.452 |
| 3 | Bagasse | CB10_04158 144h | 22.448 | -0.633 |  | Glucose | CB10_04158 144h | 25.916 | -0.452 |
| 1 | Bagasse | CB10_153.4461 24h | 24.837 | 0.906 |  | Glucose | CB10_153.4461 24h | 27.577 | 1.897 |
| 2 | Bagasse | CB10_153.4461 24h | 24.837 | 0.906 |  | Glucose | CB10_153.4461 24h | 27.577 | 1.897 |
| 3 | Bagasse | CB10_153.4461 24h | 24.837 | 0.906 |  | Glucose | CB10_153.4461 24h | 27.577 | 1.897 |
| 1 | Bagasse | CB10_153.4461 96h | 24.837 | 0.906 |  | Glucose | CB10_153.4461 96h | 27.577 | 1.897 |
| 2 | Bagasse | CB10_153.4461 96h | 24.837 | 0.906 |  | Glucose | CB10_153.4461 96h | 27.577 | 1.897 |
| 3 | Bagasse | CB10_153.4461 96h | 24.837 | 0.906 |  | Glucose | CB10_153.4461 96h | 27.577 | 1.897 |
| 1 | Bagasse | CB10_153.4461 144h | 24.837 | 0.906 |  | Glucose | CB10_153.4461 144h | 27.577 | 1.897 |
| 2 | Bagasse | CB10_153.4461 144h | 24.837 | 0.906 |  | Glucose | CB10_153.4461 144h | 27.577 | 1.897 |
| 3 | Bagasse | CB10_153.4461 144h | 24.837 | 0.906 |  | Glucose | CB10_153.4461 144h | 27.577 | 1.897 |
| 1 | Bagasse | CB10_00347 24h | 22.195 | -1.735 |  | Glucose | CB10_00347 24h | 25.417 | -0.493 |
| 2 | Bagasse | CB10_00347 24h | 22.195 | -1.735 |  | Glucose | CB10_00347 24h | 25.417 | -0.493 |
| 3 | Bagasse | CB10_00347 24h | 22.195 | -1.735 |  | Glucose | CB10_00347 24h | 25.417 | -0.493 |
| 1 | Bagasse | CB10_00347 96h | 22.195 | -1.735 |  | Glucose | CB10_00347 96h | 25.417 | -0.493 |
| 2 | Bagasse | CB10_00347 96h | 22.195 | -1.735 |  | Glucose | CB10_00347 96h | 25.417 | -0.493 |
| 3 | Bagasse | CB10_00347 96h | 22.195 | -1.735 |  | Glucose | CB10_00347 96h | 25.417 | -0.493 |
| 1 | Bagasse | CB10_00347 144h | 22.195 | -1.735 |  | Glucose | CB10_00347 144h | 25.417 | -0.493 |
| 2 | Bagasse | CB10_00347 144h | 22.195 | -1.735 |  | Glucose | CB10_00347 144h | 25.417 | -0.493 |
| 3 | Bagasse | CB10_00347 144h | 22.195 | -1.735 |  | Glucose | CB10_00347 144h | 25.417 | -0.493 |
